# Biosensors Based on the Binding Events of Nitrilotriacetic Acid–Metal Complexes

**DOI:** 10.3390/bios13050507

**Published:** 2023-04-28

**Authors:** Lin Zhu, Yong Chang, Yingying Li, Mingyi Qiao, Lin Liu

**Affiliations:** College of Chemistry and Chemical Engineering, Anyang Normal University, Anyang 455000, China; zhulin1231989@aynu.edu.cn (L.Z.); yongchang_swudc@163.com (Y.C.); liyingying2362@163.com (Y.L.); qiaomingyi2003@163.com (M.Q.)

**Keywords:** nitrilotriacetic acid, metal complexes, molecular recognition, biosensors

## Abstract

Molecular immobilization and recognition are two key events for the development of biosensors. The general ways for the immobilization and recognition of biomolecules include covalent coupling reactions and non-covalent interactions of antigen–antibody, aptamer–target, glycan–lectin, avidin–biotin and boronic acid–diol. Tetradentate nitrilotriacetic acid (NTA) is one of the most common commercial ligands for chelating metal ions. The NTA–metal complexes show high and specific affinity toward hexahistidine tags. Such metal complexes have been widely utilized in protein separation and immobilization for diagnostic applications since most of commercialized proteins have been integrated with hexahistidine tags by synthetic or recombinant techniques. This review focused on the development of biosensors with NTA–metal complexes as the binding units, mainly including surface plasmon resonance, electrochemistry, fluorescence, colorimetry, surface-enhanced Raman scattering spectroscopy, chemiluminescence and so on.

## 1. Introduction

The immobilization of biomolecules onto solid supports (e.g., electrodes, chips, quartz and substrates) or functional units (e.g., dyes, enzymes and nanomaterials) is of great importance for implementing bioassays [1,2]. A desired methodology for bioreceptor immobilization should ensure the following terms: (i) a proper orientation and uniform distribution to promise target accessibility, recognition and detectability; (ii) stability and robustness to allow flow-through assays or sequential measurement cycles; and (iii) antifouling capabilities to lower nonspecific interactions and reduce false positive signals [3]. Currently, versatile immobilization strategies have been reported, including physical adsorption based on electrostatic and hydrophobic interactions, direct chemical adsorption based on the gold–thiol binding, chemical cross-linking to functional monolayers and affinity-or linker-mediated immobilization [4,5]. The selection of an appropriate immobilization approach is dependent on the physicochemical and chemical properties of solid interfaces and target proteins as well as the intended usage scenario. For example, surface-accessible active functional groups of exposed amino acid residues, such as an amine group in lysine residue and thiol group in cysteine residue, can be utilized as the anchoring points through covalent coupling onto a pretreated solid surface [6,7,8,9]. Despite the high simplicity and stability, covalent binding may lead to the random orientation of biomolecules and the follow-up conformational change, thus decreasing the activity of bioreceptor and the partial or complete loss of its binding ability toward the target. Thus, site-specific immobilization strategies based on specific recognitions or affinity ligands are peculiarly attractive for the construction of biosensors. Typically, biotinylated antibodies have been well tethered to the avidin-modified layers via the strong avidin–biotin interactions [10,11,12]. However, the chemical modification of proteins may result in the decline inactivity and the presence of multiple siteson proteins may cause their uncontrollable orientation.

The immobilized metal ion affinity chromatography (IMAC) principle is based on the relatively strong interaction between transition metal cations (e.g., Cu^2+^, Ni^2+^, Zn^2+^ and Co^2+^) and accessible metal-binding amino acid residues (e.g., cysteine, histidine and tryptophan) [13,14,15]. It is originally applied to the purification of proteins containing histidine residues on the surface with the equal strength of “bio-specific” interactions. A multitude of metal-chelating ligands with different denticities have been fixed on the solid support matrixes changed from agarose gels to rigid silica particles. In this process, the ligands act as Lewis bases to bind divalent transition metal ions, such as tridentate iminodiacetic acid, tetradentate nitrilotriacetic acid (NTA) and pentadentatetris(carboxymethyl)ethylene diamine [16,17]. The entrapped metal ions serve as Lewis acids and the remained unoccupied coordination sites can further ligate to the imidazole moieties of hexahistidine (His_6_) tags. As coordination-bonding-based artificial receptors, the ligands exhibit different IMAC protein separation efficiencies based on the number and conformation of chelation sites [18,19]. Among them, the tetradentate ligand of NTA can be chelated with bivalent transition metal cations (e.g., Cu^2+^, Ni^2+^, Zn^2+^ and Co^2+^) to form a hexagonal complex and two unoccupied coordination sites remain for the further ligation to the imidazole moieties of His_6_ tag. Nowadays, NTA-based IMAC is one of the most promising approaches for reversible and controlled protein immobilization [3,20].

Some characteristic groups of biomolecules can be used as the anchor sites or affinity tags for the coupling of them onto the transducer surfaces with preserving activity [21]. As one of the smallest and most commonly used affinity elements, His_6_ tag can be readily fused to the N- or C-terminal of a synthetic peptide or recombinant protein without influence on the target-binding activity [22]. Moreover, the immobilized His_6_-tagged proteins can be easily displaced by competing coordinators (e.g., ethylenediaminetetraacetic acid (EDTA) or imidazole) under mild conditions, realizing the elution of targets and the regeneration of solid surfaces and materials [23,24]. Furthermore, histidine-rich proteins with unusually high histidine contents can also interact with NTA–metal complexes [25,26]. The dysregulation of these proteins has been associated with several diseases, including liver cirrhosis, cancer, asthma and pulmonary disease [27,28]. Wright’s group has reported a series of novel works for *Plasmodium falciparum* histidine-rich protein 2 (*pf*HRP-II) extraction and detection using NTA–metal complexes [29,30,31]. Thus, the broad arrays of chelators modified on various materials, including NTA, polydopamine, 1-acetato-4-benzyl-triazacyclononane and chitosan, have been popularly used to extract and immobilize biomolecules from real samples while retaining bioactivity for further research [32,33,34,35,36,37,38,39]. Currently, countless NTA-functionalized molecules and materials are commercially available for protein immobilization and site-specific labeling, including fluorescent dyes, lipids, antibodies, peptides, magnetic beads and gold nanoparticles [40]. Considering their great potential, the applications of NTA–metal complexes in different fields have been summarized in several reviews [41,42,43,44]. For instance, Wieneke et al. reviewed the development of multivalent chelators for in vivo protein labeling [45]. You et al. summarized the progress of multivalent chelators for spatially and temporally controlled protein functionalization [46]. Lόpez-Laguna et al. provided comprehensive insights on the emerging biotechnology of histidine-rich peptides [47]. However, no systematic reviews currently focus on the advancement of biosensors with NTA–metal complexes as the binding units. To maintain the theme of this review, herein, we summarized the current developments of biosensors based on the binding events of NTA–metal complexes. We classified the developments of such biosensors according to the detection techniques, including surface plasmon resonance (SPR), electrochemistry, fluorescence, colorimetry, surface-enhanced Raman scattering spectroscopy (SERS), chemiluminescence and so on. Moreover, future challenges and research trends for NTA–metal complexes-based bioassays are briefly discussed.

## 2. NTA–Metal Complexes−Based Biosensors

There are several important factors for the modulation of the binding affinity and the constant between NTA–metal complexes and biomolecules. The choice of the NTA–metal-based system for a particular application is critical for the efficient immobilization and detection of biomolecules [48]. The reversible character is favorable for protein purification and a higher affinity interaction is desired in the case of protein labeling in vitro and in live cells. The length, number and position of His_6_ tag may influence the purification and immobilization of recombinant proteins [49,50,51]. In this aspect, Knechtet al. investigated the binding properties between several different series of oligohistidines as well as mixed oligohistidines/oligoalanines and Ni^2+^–NTA by SPR experiments [52]. The results suggested that His_6_ tag possessed an equilibrium dissociation constant (K_D_) of 14 ± 1 nM, and the highest affinity of the peptides and two His residues separated by either one or four residues are the preferred binding motifs. Although a longer histidine tag (e.g., His_8_ or His_10_) can achieve higher purity efficiency, it may cause the inhibition of protein functions and require a higher concentration of imidazole to elute. In addition, Madoz-Gúrpide et al. suggested that the orientation of enzyme ferredoxin/NADP^+^ reductase on the surface of NTA–Cu^2+^ complexes-functionalized electrode could be tuned by adjusting the position of a histidine pair (His–X_3_–His) in α-helices [53]. Schröper et al. investigated the effect of His_6_ tag-based affinity-binding strategy on the immobilization of redox protein horse heart cytc on the gold electrode surface [54]. It was found that cytc with C-terminal His tag exhibited the strongest redox signal due to the proximity between the His_6_ tag and the intramolecular electron transfer pathway. Moreover, Khan et al. found that double-His_6_ tags separated by an 11-amino acid spacer exhibited at least one order of magnitude stronger binding affinity to Ni–NTA-modified surfaces, compared with the single-His_6_ tag or two single-His_6_ tags at both the N- and C-terminals [55].

An individual metal–NTA–His_6_ complex shows relatively low stability and affinity (K_D_ = 1 × 10^−5^ M) [56]. The binding stability can be enhanced by increasing the surface density of NTA [57,58]. Multivalent chelators such as di- (2.7 × 10^−7^ M), tri- (2 × 10^−7^ M) or tetra-NTA (4 × 10^−8^ M) derivatives have also been designed to achieve a high density of chelators as binding sites, thus enhancing the NTA-based surface stability [59,60,61,62,63]. For instance, Lata et al. demonstrated that an increasing number of NTA moieties could lead to a substantial increase in binding stability, achieving a subnanomolar affinity [64]. You et al. reported the application of multivalent chelators for high-affinity and spatially and temporally controlled the recognition and functionalization of His_6_-tagged proteins [46]. Moreover, other irreversible covalent interactions were combined with the reversible coordination interaction, including photochemical reaction, amine coupling reaction and epoxide chemistry [65,66,67,68,69]. However, these approaches typically require complicated synthetic processes and lack high specificity.

The chelators exhibit a different affinity for bivalent metal ions (Cu^2+^ > Ni^2+^ > Zn^2+^ ≥ Co^2+^) and distinctive specificity (Co^2+^ > Zn^2+^ > Ni^2+^ > Cu^2+^) toward His_6_-tagged proteins, which may affect their utilization in practical applications [70,71]. Compared with the carboxyl self-assembled monomer (SAM), that of Ni–NTA can pattern His_6_-tagged biomolecules in a higher immobilization capacity and binding activity, improving the detection sensitivity [72]. However, the sensing surface may suffer from slow and continuous dissociation of immobilized biomolecules due to the low affinity and stability between the His-tagged biomolecules and NTA mediated by Ni^2+^ and other metal ions [73]. Moreover, low pH, reductants and chelators, such as EDTA and imidazole in matrices, may quickly disturb the NTA–metal complexes. Cu^2+^ shows the greatest affinity, which can be used to isolate low abundant proteins from crude lysates, followed by other purification steps. In addition, the conversion of bivalent metal ions to different metal oxidation states is one of the alternative strategies to address those shortcomings and maintain the benefits of His_6_-tagged protein immobilization [74]. For example, Spatz’s group developed the Co^3+^-mediated, stable and kinetically inert interaction between His_6_ tag and NTA for a permanent, oriented and specific protein immobilization [75,76]. In this strategy, the formation constant of exchange-inert Co^3+^ complexes is higher than that of conventional Co^2+^ and Ni^2+^ complexes, which is resistant toward competitive chelators and washing off over time.

In case of the NTA–Ni^2+^–His_6_ tag system, the stability constants of four complexes should be considered, including Ni^2+^/His_6_-tagged protein, Ni^2+^/NTA (K_D_ = 1.8 × 10^−11^ M), Ni^2+^/imidazole (K_D_ = 9.8 × 10^−4^ M) and Ni^2+^/EDTA (K_D_ = 4 × 10^−19^ M) [52]. Taking advantage of the differences between the adjustable dissociation constants, the captured His_6_-tagged proteins can be eluted by imidazole under mild conditions, resulting in Ni^2+^ ions to remain bound to NTA. In the context of protein purification, the addition of imidazole can improve the selectivity of NTA–metal complexes toward His_6_-tagged proteins. Based on the differences between the dissociation constants of Ni^2+^/EDTA and Ni^2+^/NTA, the surface can be regenerated by completely removing Ni^2+^ ions with EDTA and then followed by repeatedly loading the chelators with Ni^2+^ ions [52]. The regeneration of sensing surfaces can make the ligand density similar with that for the incubation or injection of each analyte, which is helpful for the conventional analysis [77].

The compatibility of NTA with chemical conjugation protocols can enable versatile and efficient surface chemistries for the robust and reproducible immobilization of His_6_-tailed biomolecules on different solid surfaces and nanomaterials. SAMs of NTA–metal chelators can endow the interface with desired properties. At present, various strategies have been reported to modify the interface with NTA–metal chelators for the deposition of His_6_-tagged biomolecules [78,79,80]. For example, NTA moieties can be functionalized with different alkyl thiols to form metal-chelating layers on gold electrodes [81,82,83]. However, the synthesis of the alkane thiol chelators was complicated and the formed monolayer was less well-ordered. To overcome these difficulties, NTA modified with an amino group can be covalently tethered onto the carboxyl-terminated SAM preformed on the electrode through the 1-(3-dimethylaminopropyl)-3-ethylcarbodiimide (EDC)/N-hydroxysuccinimide (NHS)-activated amine coupling reaction [58,84,85]. In addition, Haddour et al. reported that the pyrrole monomer modified with NTA could be electrochemically polymerized into a conductive poly(pyrrole)-NTA film for the reversible oriented immobilization of His_6_-tagged proteins [86]. NTA conjugated with pyrene can attach onto carbon-based nanomaterials (e.g., carbon nanotubes and graphene) deposited on the interface via π-stacking interactions between pyrene derivatives and the materials, which could be reinforced by electropolymerization [87,88,89,90].

### 2.1. SPR Biosensors

SPR technique can determine the binding affinity and kinetics between ligands and receptors, which has been widely used in the monitoring of various biological recognition events in real time. The rational fabrication of biorecognition interface is responsible for the reliability and accuracy of SPR assays [91]. Among various immobilization strategies, NTA–metal complex-aided approach can facilitate the immobilization of His_6_-tagged bioreceptors on SPR platform in a site-specific and oriented manner [92,93,94,95]. For example, thiol-functionalized NTA can be tethered on the gold-based SPR chip [96]. However, the oxygen sensitivity of thiol species may cause the degradation of the chemisorbed SAM during storage. For this consideration, NTA could be coupled to polymer brushes which were pre-immobilized on the chips for the construction of protein-resistant interfaces [97,98].

**Figure 1:**
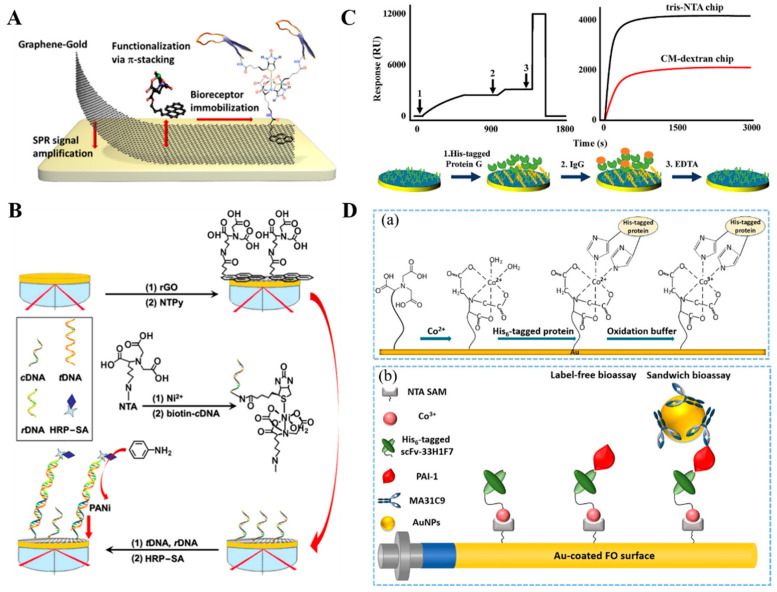
(**A**) Schematic illustration of the functionalization of the graphene layer via π-stacking of pyrene-NTA followed by electropolymerization for the reinforcement of the layer [101]. Copyright 2015 American Chemical Society. (**B**) Schematic illustration of the noncovalent functionalization of rGO for sensitizing SPR-based DNA sensing synergistically with biocatalytic polymerization [102]. Copyright 2017 Elsevier. (**C**) Schematic illustration of trisNTA-based rapid and regenerable SPR determinations of biomarker concentration and biomolecular interaction [113]. Copyright 2021Elsevier. (**D**) Schematic illustration of (**a**) the immobilization steps of His_6_-tagged protein on Au-coated surface by Co^3+^–NTA strategy and (**b**) FO-SPR based bioassay of PAI-1 using Co^3+^–NTA for bioreceptor immobilization [70]. Copyright 2020 American Chemical Society.

His_6_-tagged biomolecules can be immobilized on NTA-functionalized chips for the sensitive and accurate investigation of the interaction between bioreceptor and analyte [99,100]. The integration of single-layer graphene with gold chip can improve the sensitivity of SPR biosensors. Singh et al. developed a SPR immunosensor by growing graphene on the chip surface through chemical vapor deposition, which is different from that of graphene oxide, reduced graphene oxide (rGO) or grapheme decorated metal nanoparticle-based platforms (Figure 1A) [101]. In this study, graphene was modified with the film of ploypyrrole-NTA or pyrene-NTA, followed by the immobilization of biotinylated cholera toxin as the bioreceptor unit via the effective NTA–Cu^2+^/biotin system for antibody detection. The result demonstrated that the ultrathin functional layer formed by the π-stacking interaction of pyrene-NTA and the subsequent electropolymer achieved the best detection performance (Table 1). With the similar immobilization system, Yuan et al. reported SPR-based DNA assays using an NTA–Cu^2+^-covered graphene-modified chip to immobilize a biotinylated DNA capture probe, in which the enzymatic catalysis was integrated into SPR assay for signal amplification (Figure 1B) [102]. In this study, nickel-chelated pyrene-NTA was tethered onto the rGO-modified chip to immobilize biotin-labeled capture DNA. After the hybridization between capture DNA, target DNA and reporter DNA, HRP-tagged reporters could catalyze the conversion of aniline into polyaniline precipitation, resulting in great signal amplification via the mass-effect. However, the gradual dissociation of His_6_-tagged proteins may result in an unstable baseline, adversely influencing the accurate analysis.

To increase the binding stability, Wang et al. reported the SPR detection of small molecule binding events by integrating the His_6_–Ni^2+^ coordination and the amine coupling reaction to covalently affix His_6_-tagged proteins [103,104]. Although proteins exhibit amore uniform orientation and a higher density through this immobilization approach, the chip surface would not be regenerated due to the covalent linkage. Double- or triple-His_6_ residues could be added into the sequence of proteins for enhancing the attachment [55,105,106]. However, the increased cost and complexity in expressing proteins with double- or triple-His_6_ residues will present a disagreeable problem. To overcome this shortcoming, trisNTA-functionalized polymers, such as poly-L-lysine graftpoly(ethyleneglycol) polymer and dextran, were utilized to stably yet reversibly bind His_6_-tagged or biotin-labeled proteins for the investigation of different protein bindings and interactions [107,108,109,110,111,112]. With a trisNTA–Ni^2+^-covered chip, Liu et al. developed a SPR biosensor for the detection of biomarkers in body fluids (Figure 1C) [113]. It was found that the His_6_-tagged proteins attached on the chip surface could be readily regenerated by changing the pH of EDTA solution. Alternatively, Spatz’s group reported a novel surface functionalization strategy by using Co^3+^ ion as the mediator between NTA and His_6_-tagged protein [76,114]. In this approach, Co^2+^ ion in the complex was oxidized to Co^3+^ in situ by H_2_O_2_. Compared with Co^2+^ and Ni^2+^ complexes, Co^3+^ complexes exhibit exchange-inert property, higher association and lower dissociation rate constant under the similar coordination environments [114]. Thus, Co^3+^ complexes have been employed to immobilize His_6_-tagged proteins for bioassays, such as QCM, biolayer interferometry and fluorescent assays [99,115,116,117,118,119]. Notably, Lammertyn’s group used NTA–Co^3+^-modified surface to design fiber optic (FO)-SPR biosensors (Figure 1D) [70]. The analytical performances, including immobilization efficiency, surface coverage, reproducibility, stability and specificity, were investigated with plasminogen activator inhibitor-1 (PAI-1) as the model example. His_6_-tagged anti-PAI-1 antibody fragment (scFv-33H1F7) was used as the receptor and anti-PAI-1 monoclonal antibody (MA-31C9)-modified AuNPs were used as the recognition elements for signal amplification.

### 2.2. Electrochemical Biosensors

**Figure 2:**
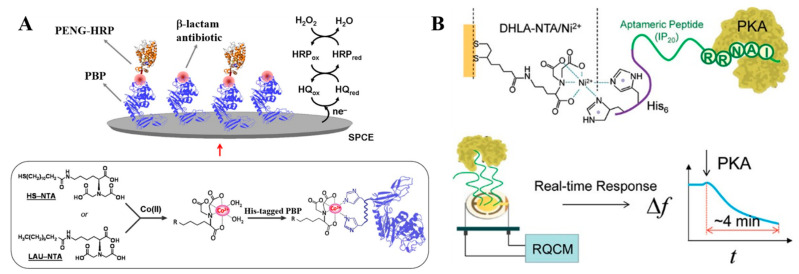
(**A**) Schematic illustration of the development of the affinity biosensor involved in the immobilization of the recombinant His_6_-tagged PBP by using Co^2+^−NTA-modified SPCEs [133]. Copyright 2013 American Chemical Society. (**B**) Schematic illustration of the aptameric peptide (IP_20_)−PKA conjugate as a sensing platform to monitor kinase [134]. Copyright 2012 American Chemical Society.

Electrochemical biosensors can measure the target concentration by monitoring the change of current, potential, conductance or impedance at a solid electrode [120,121]. The electrode modifiers can significantly affect the performances of electrochemical biosensors. The assembly of redox enzymes in a proper alignment is particularly critical for the realization of fast electron transfer between the electrode surface and the redox center of enzyme, retaining the catalytic and regulatory property of protein [122,123,124]. The SAMs of NTA–metal chelators on the electrode surface can allow for the well-controlled and reversible immobilization of a wide range of His_6_-tagged enzymes via the specific affinity binding, including horseradish peroxidase (HRP), alkaline phosphatase (ALP), laccase and glucose oxidase and nitrate reductase [125,126,127,128,129,130]. For instance, Blankespoor et al. fabricated a dense monolayer of NTA–Cu^2+^ complexes on the surface of a carbon electrode for the immobilization of His_6_-tagged HRP and realized the electrochemical reduction of H_2_O_2_ in the presence of an artificial redox mediator [131]. Wang et al. reported the immobilization of superoxide dismutase (SOD) on the NTA–Ni^2+^-modified electrode for the in vivo detection of O_2_^•−^ in a rat brain, demonstrating that the direct electron transfer of SOD was greatly enhanced by the NTA–Ni^2+^ complexes (Table 1) [132]. Conzuelo et al. reported the competitive detection of *β*-lactam antibiotics using an NTA–Co^2+^-modified electrode to immobilize the recombinant bacterial penicillin binding protein (PBP) (Figure 2A) [133]. In this study, HRP-labeled specific tracer (PENG-HRP) was used as the signal label for the competitive binding and hydroquinone (HQ) was used as the redox mediator for the catalytic oxidation of H_2_O_2_.

Aptamers including DNA/RNA and peptide possess several intrinsic properties, such as excellent structure flexibility, high specificity and affinity, good target diversity and ease of synthesis. The immobilization of His_6_-tagged aptamers on an NTA–metal complex-modified electrode can produce a highly oriented aptamer assembly to prevent the nonspecific adsorption [135]. Cosnier’s group utilized poly(pyrrole-NTA) film to immobilize His_6_-tagged aptamer in the presence of Cu^2+^ ions for a label-free impedimetric detection of thrombin and bisphenol-A, respectively [136,137]. Quartz crystal microbalance (QCM) is a simple and direct electrochemical method to study molecular interactions. Xu et al. reported the QCM-based detection of protein kinase A (PKA) with a His_6_-tagged peptide inhibitor of IP_20_ as the aptamer-mimicking biorecognition element, in which the aptameric peptide was immobilized on the NTA–Ni^2+^-covered quartz electrode (Figure 2B) [134]. The kinase concentration could be sensitively determined based on the frequency response of the QCM crystal. In addition, Zaitouna et al. developed an electrochemical biosensor for Ara h 2 antibody detection using the NTA–Ni^2+^ SAM to immobilize the His_6_-tagged and methylene blue (MB)-labeled peptide [138]. The detection performance of NTA–Ni^2+^SAM-based assay was better than that of the biosensor based on the conventional immobilization with a thiolated peptide.

**Figure 3:**
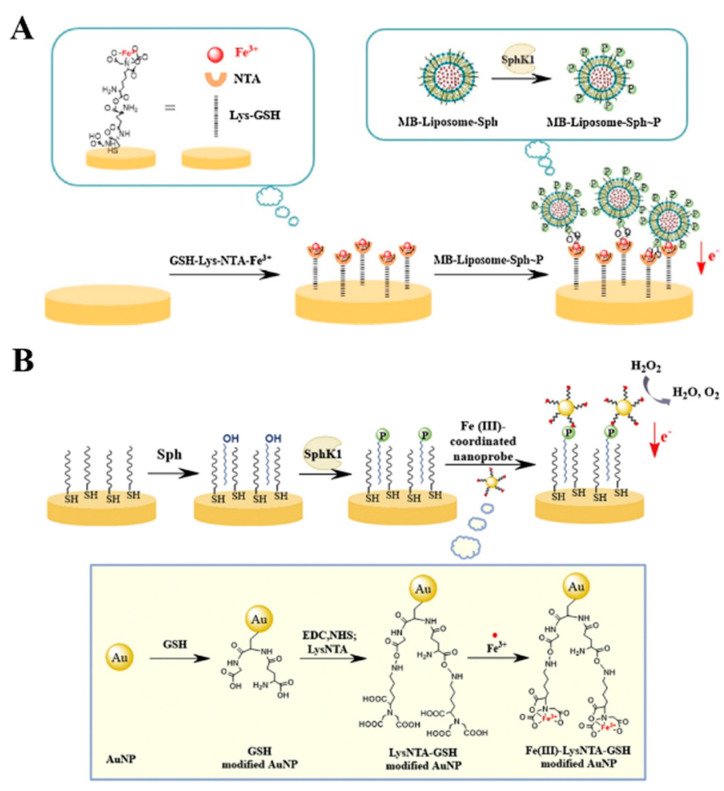
(**A**) Schematic illustration of the principle and fabrication procedures for lipid kinase activity based on liposome-assisted electrochemical assay [142]. Copyright 2017 Elsevier. (**B**) Schematic illustration of SphK1 activity assay based on the bifunctional NTA–Fe^3+^ complex-modified AuNPs [143]. Copyright 2016 Elsevier.

Aside from His_6_ tag, biotinylated biomolecules can bind with NTA–metal complexes because three potential binding sites (carboxylate, thioether and ureido groups) of biotin can coordinate with a bivalent metal cation in the NTA chelate [88,139]. For this view, Bauret al. reported the immobilization of biotinylated GOx and polyphenol oxidase on the Cu^2+^-chelated poly(pyrrole-NTA) film for the amperometric detection of glucose and catechol, respectively [140]. Meanwhile, Palomar et al. developed an impedimetric immunosensor for the determination of an anticholera toxin antibody by the immobilization of biotin-labeled cholera toxin B subunit on the Cu^2+^-chelated poly(pyrrole-NTA) [141]. The coordinated metal ions in an NTA complex can also bind specifically to phosphorylated biomolecules. Gao et al. reported an electrochemical assay for sphingosine kinase 1 (SphK1) detection using NTA–Fe^3+^ complex to recognize phosphorylated lipids on liposomes [142]. As presented in Figure 3A, liposome was used to embed a substrate of SphK1 in lipid layer through hydrophobic interaction and encapsulate electroactive MB molecules. After the catalytic reaction on the membrane, the reacted liposomes were captured by the NTA–Fe^3+^ complex-modified sensing electrode, and the abundant MB molecules in liposome could generate a strong electrochemical signal, thus reflecting the kinase activity.

SAM of NTA assembled on the electrode can coordinate with metal ions for electrochemical detection by stripping voltammetry. For instance, Kerekovic et al. investigated the copper(II) binding capacity of an NTA-modified gold electrode via adsorption transfer stripping voltammetry [144]. The results showed that Cu^2+^ ions in the chelates could be directly determined by adsorption transfer stripping voltammetry without an electrochemical accumulation step. Meanwhile, Sasaki et al. developed an electrical assay for the on-site detection of Cu^2+^ ions based on the SAMs of NTA-modified organic thin-film transistor [145]. The coordination of Cu^2+^ ions with NTA could induce the potential shift of the extended-gate, generating an observed change in the drain current. Moreover, Fe^3+^ in the chelating condition retains the ability to catalyze the electrochemical redox of H_2_O_2_. Gu et al. reported a bifunctional NTA–Fe^3+^ complex-based nanoprobe for the electrochemical detection of SphK1 activity [143]. As displayed in Figure 3B, gold nanoparticles (AuNPs) were employed to carry NTA–Fe^3+^ complexes to recognize phosphorylated sites of substrates. Then, multiple NTA–Fe^3+^ complexes and AuNPs catalyzed the decomposition of H_2_O_2_, largely amplifying the catalytic amperometric response.

### 2.3. Fluorescence Biosensors

Fluorescence assays are the most commonly used optical methods to determine molecular interaction, mobility and conformational change. NTA moieties have been linked to peptide substrates or modified with fluorescent units for fluorescent bioassays and bioimaging [146,147,148,149]. For example, Kim et al. used NTA–Ni^2+^ complex-modified tetramethylrhodamine (TMR)-doped SiO_2_nanoparticles to label a bacterial lysate containing estrogen receptor R ligand binding domain [150]. SiO_2_ NPs could improve the sensitivity and limit the fluorescence quenching of dyes by external nickel ions.

**Figure 4:**
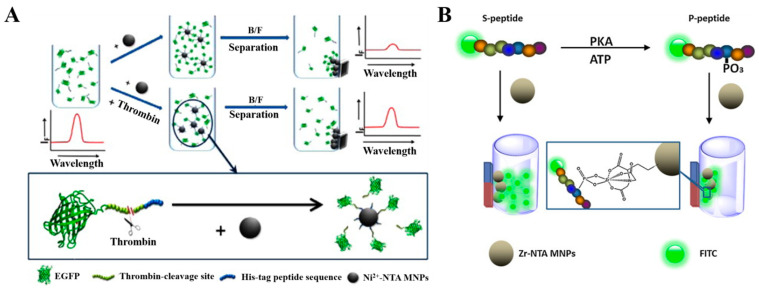
(**A**) Schematic illustration of the strategy using Ni^2+^–NTA MNPs and the recombinant EGFP to detect the activity of thrombin [151]. Copyright 2013 Elsevier. (**B**) Schematic illustration of the fluorescence kinase activity assay based on Zr–NTA MNPs enrichment [152]. Copyright 2013 Elsevier.

Magnetic nanoparticles (MNPs) or magnetic beads (MBs) can be facilely manipulated with an extra magnetic field. They have been widely used in protein/peptide isolation after modification with NTA–metal complexes. Thus, NTA–metal–coated MBs or MNPs have been employed to separate fluorescently labeled peptides from a homogeneous solution (Table 1), leading to the change of fluorescence intensity. For example, Wang et al. reported a label-free fluorescent method for the detection of thrombin activity based on a His_6_-tagged recombinant green fluorescence protein (EGFP) and Ni^2+^–NTA-coated MNPs [151]. As shown in Figure 4A, EGFP with a thrombin cleavage site and a His_6_ tag at the N-terminal could be enzymatically cleaved by thrombin, thus resulting in the release of His_6_ tag with the inability to attach NTA–Ni^2+^-coated MNPs. After magnetic separation, the fluorescence intensity of EGFP in the solution is positively related to the activity of thrombin. However, the high cost, large size and pH sensitivity may limit the application of the method for protease assays. In addition, Tan et al. developed a fluorometric method for the detection of protein kinase activity based on the adsorption between NTA–Zr^4+^ MNPs and phosphorylated peptides [152]. As shown in Figure 4B, the fluorescein isothiocyanate (FITC)-conjugated substrate peptide was phosphorylated by protein kinase. The phosphorylated product could adsorb on the surface of NTA–Zr^4+^ MNPs via the chelation of Zr^4+^ and phosphate. After magnetic separation, the fluorescence intensity of the solution evidently decreased, which was indicative of the activity of protein kinase.

**Figure 5:**
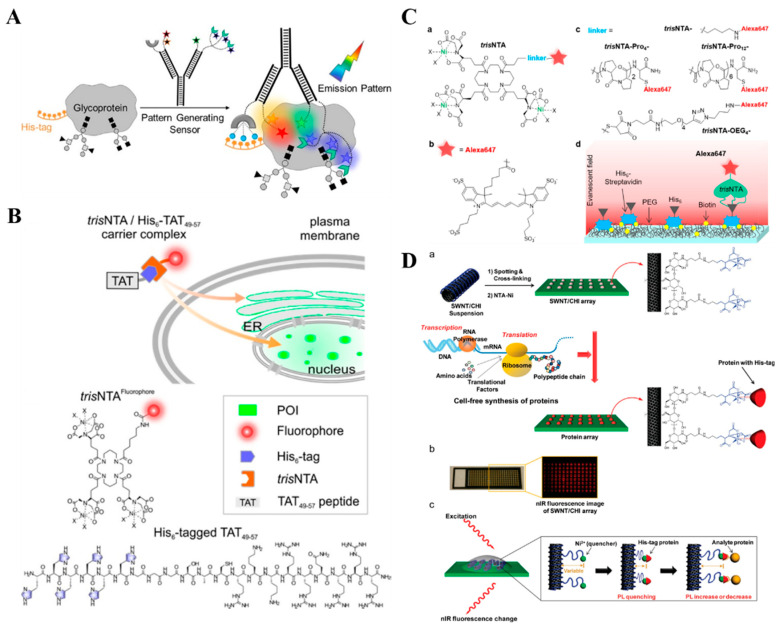
(**A**) Schematic illustration of glycoform differentiation by a targeted, self-assembled, pattern-generating protein surface sensor [165]. Copyright 2020 American Chemical Society. (**B**) Schematic illustration of live-cell labeling of His-tagged proteins in distinct cellular compartments using a cell-penetrating noncovalent *tris*NTA carrier complex formed by fluorescent trisNTA and His_6_-tagged TAT_49−57_ [166]. Copyright 2018 American Chemical Society. (**C**) Schematic illustration of: (**a**) the structure of *tris*NTA construct, (**b**) structure of Alexa647, (**c**) structure of the linkers used for *tris*NTA–Alexa647; *tris*NTA–Pro12-Alexa647; *tris*NTA–Pro4-Alexa647 and *tris*NTA–OEG_4_-Alexa647 constructs. (**d**) Schematic illustration of the single-molecule experiment used to evaluate the photostability of *tris*NTA constructs [170]. Copyright 2018 American Chemical Society. (**D**) Schematic of a label-free protein array based on fluorescent NTA–Ni^2+^ complex-modified SWNT [173]. (**a**) Array fabrication using SWNT/CHI and in situ generation of individually addressed capture proteins using cell-free protein synthesis for label-free optical detection of protein interactions. A SWNT/CHI suspension is spotted on glass and functionalized with Ni-NTA to bind His-tag-containing capture proteins. Cell-free extract and PCR amplified DNA coding for each protein were added to each spot for protein expression and in situ immobilization. (**b**) Optical and NIR fluorescence image of the SWNT/CHI array. (**c**) Signal transduction mechanism for label-free detection of protein-protein interactions: a NIR fluorescence change from the SWNT occurs when the distance between the Ni2þ quencher and SWNT is altered upon analyte protein binding. Copyright 2011 American Chemical Society.

Organic fluorophores have been extensively used to label proteins in vivo through different chemically and biologically labeling techniques without changing the structure and disrupting the normal function of proteins. NTA has been used as a chemical recognition unit to modify fluorescent probes for labeling His_6_-tagged proteins or peptides at a specific site via reversible metallochelate coupling of metal ion and His_6_ tag. A few organic dyes have been conjugated with NTA complexes to label His_6_ tags, such as fluorescein, perylene and Atto488 [56,153,154,155,156,157]. Typically, Glymenaki et al. synthesized three different porphyrin−NTA dyads and successfully employed them to label different His_6_-containing peptides [158]. Lata et al. modified different fluorophores with trisNTA groups for the selective labeling of proteins in cell lysates and on the surface of living cells [159]. Gatterdam et al. prepared several NTA-based multivalent chelators with linear, cyclic and dendritic scaffolds, respectively, and compared their performances on the labeling of cellular His_6_-tagged proteins [160]. The results demonstrated that the cyclic trisNTA chelator exhibited the highest affinity and kinetic stability. Furthermore, Uchinomiya et al. reported a site-specific covalent labeling of His_10_-tagged proteins [161]. In this study, the interaction between His-tag and NTA–Ni^2+^ facilitated the nucleophilic reaction between ahistidine residue in His_10_ tag and the electrophilic tosyl group in the NTA–Ni^2+^ probe by the proximity effect. Different from the probes that merely labeled proteins, dye–NTA conjugates designed by Margulies and coworkers could monitor the change on protein surface by altering the intensity or wavelength of emission upon binding [162,163,164]. Peri-Naor et al. used the DNA probe modified with both His tag and boronic acid group to develop targeted, pattern-generating and protein surface sensors (Figure 5A) [165]. The method can be used to discriminate between the distinct glycoform populations and identify the glycosylation states of therapeutic proteins.

The poor ability of NTA-based fluorescent probes to penetrate cell membrane may significantly limit the applications in the study of intracellular proteins and subcellular organelles. To facilitate the identification of proteins in living cells, Wieneke et al. designed a cell-penetrating multivalent *tris*NTA chelator of carrier complexes to label protein of interest (POI) based on the cell-penetrating peptide (CPP) [166]. As shown in Figure 5B, Ni^2+^-loaded and fluorophore-modified *tris*NTA could bind to His_6_-tagged HIV TAT_49−57_. After being delivered into the cytosol and nucleus, the *tris*NTA preferentially interacted with His_10_–POI, thus resulting in the release of the carrier peptide. In addition, Zhang et al. synthesized a Nap-G/Biotin/ANA-FFpYGK-NTA–Ni^2+^ probe consisting of an NTA–Ni^2+^ group, a self-assembling peptide FFpY and a hydrophobic group [167]. After the hydrolysis is catalyzed by ALP, the product could self-assemble into nanofibers and enter the cells with an increased efficiency to label His_6_-tagged proteins.

In single-molecule fluorescence imaging, it is important to improve the photostability of fluorophores by using solution additives/photostabilizers or directly conjugating the photostabilizer to the fluorophores, leading to the quenching of photodegradation-involved transient intermediates. It has been documented that Ni^2+^ ion is an efficient photostabilizing agent through a physical route to quench the triplet excited state of some fluorophores [168,169]. For example, Glembockyte et al. designed a *tris*NTA Alexa647 fluorophore as a self-healing dye for single molecule fluorescence imaging [170]. As illustrated in Figure 5C, four different Alexa647-labeled *tris*NTA probes were synthesized with various length and rigidity of linkers. After the complexation of three Ni^2+^ ions, the photostability of Alexa647 was significantly enhanced due to the closer proximity between Ni^2+^ions and fluorophores. Moreover, *tris*NTA in the fluorophores also acted as a handle to specifically label His_6_-tagged POI for single-molecule imaging.

Transition-metal ions with paramagnetic nature can quench the fluorescence of molecules and nanomaterials with a distance-dependence property [171,172]. Ahn et al. reported the label-free, single-protein detection based on a near-infrared fluorescent NTA–Ni^2+^ complex-modified single-walled carbon nanotube (SWNT) [173]. As shown in Figure 5D, when the His_6_-tagged proteins were bound to the complex, the fluorescence intensity was reduced due to the decrease in the intermolecular distance between Ni^2+^ and SWNT. After the addition of target protein (antiHis_6_-tag antibody), the fluorescence signal increased based on the analyte–protein interaction. Based on this platform, Ahn et al. investigated glycan–lectin binding for glycan profiling [174].

**Figure 6:**
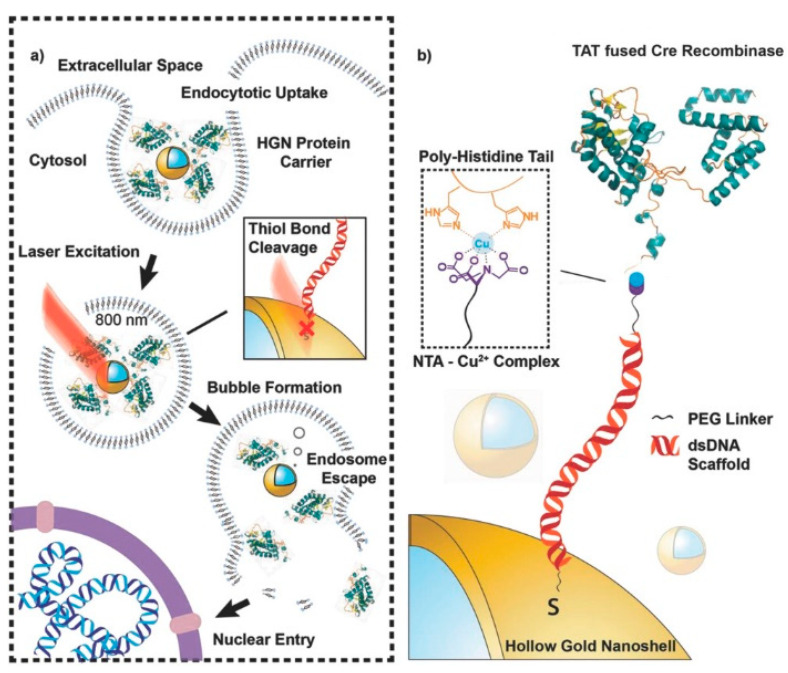
Schematic of (**a**) a light-activated delivery of gene editing enzymes, such as Cre recombinase by HGN-mediated release by NIR laser irradiation and (**b**) assembly of a TAT peptide fusion of Crerecombinase on HGN surfaces by a modular handle based on the affinity of polyhistidine tags to a NTA–metal complex presented by a double-stranded DNA scaffold [176]. Copyright 2018 WILEY-VCH.

NTA chelator can also be used to immobilize drugs and biomolecules on the surface of nanomaterials for targeting delivery. For instance, the *tris*NTA-modified graphene oxide has been used to load His_10_-tagged EGFP for the delivery of drugs into cells [175]. Morales et al. developed a light-activated genome editing platform by monitoring the release of enzymes from hollow gold nanoshell (HGN) nanocarriers [176]. As shown in Figure 6, Crerecombinase, a protein fusion with a TAT internalization peptide segment, was tested. The protein was immobilized on the HGN modified with NTA-labeled dsDNA in the presence of Cu^2+^. A red fluorescence was observed after the release of Crerecombinase.

### 2.4. Colorimetric Assays

Colorimetric assays have attracted intensive attention due to their low cost and high simplicity. Such methods do not require expensive or sophisticated instrumentation because the color change can be read by the naked eye. Enzymes can efficiently catalyze various chromogenic reactions for colorimetric assays [177,178]. For example, a phosphotriesterase (PTE) trimer can hydrolyze the substrate organophosphate paraoxon to produce faint yellow *p*-nitrophenol. However, the stability and activity of enzymes are not sufficient for usage in the development of portable sensing devices. Medintz’s group demonstrated that the stability and activity of PTE were obviously enhanced when it was adsorbed onto NTA–Ni^2+^ complex-modified AuNPs [179]. For this view, they developed a colorimetric method for the detection of organophosphates with PTE–NTA–Ni^2+^-AuNPs [180].

Peptide-based colorimetric assays have been widely developed to evaluate different protease activities. The signal reporter-modified His_6_-tagged peptide can be immobilized on the solid surface or nanoparticles (Table 1). In the presence of target protease, peptide was enzymatically hydrolyzed and the release of signal reporter would result in the change of solution color. Moss et al. designed an enzyme-based amplification system for the colorimetric detection of proteases [181]. HRP-conjugated His_6_-tagged substrate peptides were attached onto NTA–Ni^2+^–modified MBs. In the presence of target enzymes matrix metalloproteinase 2 or disintegrin and metalloproteinase 8, the peptide was cleaved to release HRP into solution. After magnetic separation, HRP in the unreacted peptide on the MBs was quantified by a standard HRP color assay with 3,3′,5,5′-tetramethylbenzidine (TMB) and H_2_O_2_ as the substrates. However, the high cost and complicated cross-linking procedures of the enzyme-conjugated substances may limit the applications of the colorimetric assays.

**Figure 7:**
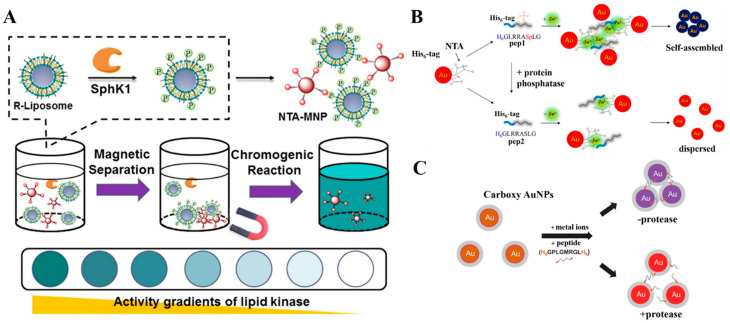
(**A**) Schematic illustration of the principle and assay procedures of the magneto-colorimetric assay for SphK1 activity [182]. Copyright 2018 American Chemical Society. (**B**) Schematic illustration of colorimetric assay for protein phosphatase activity based on AuNPs and His_6_-tagged phosphopeptides in the presence of Zn^2+^ [183]. Copyright 2015 American Chemical Society. (**C**) Schematic illustration of the colorimetric assay for protease activity based on metal-induced self-assembly of AuNPs [184]. Copyright 2013 Elsevier.

To enhance the sensitivity of colorimetric methods, nanomaterials can be used as the carriers to load signal molecules in a high loading efficiency. Under the external stimulus, such as pH, light and surfactants, signal molecules will be rapidly released, generating a significant colorimetric response for signal amplification. For this consideration, Gao et al. reported an integrated magneto-colorimetric method for the assay of lipid kinase (SphK1) activity using NTA-modified MNPs and TMB-loaded liposomes [182]. As shown in Figure 7A, lipid substrates anchored on liposomes were phosphorylated by SphK1. NTA–Fe^3+^–MNPs could specifically bind to the phosphate sites on liposomes. After magnetic separation, TMB molecules were released from the liposomes and then oxidized by H_2_O_2_ under the catalysis of NTA–MNPs, producing a colorimetric signal for the visual detection of SphK1 activity.

The aggregation/disaggregation of AuNPs can result in a detectable color change due to the surface plasmon coupling. Based on this unique property, several colorimetric assays based on AuNPs and NTA–metal complexes have been reported for clinical diagnosis and environmental protection [31,185,186]. For example, Lee et al. reported the AuNPs-based colorimetric assay of protein phosphatase activity through Zn^2+^–phosphate interaction (Figure 7B) [183]. The His_6_-tagged peptide substrates could bind to NTA–Ni^2+^-covered AuNPs by specific coordination. After phosphorylation, the peptide could trigger the aggregation of AuNPs in the presence of Zn^2+^ ions. In addition, Kim et al. found that the carboxy AuNPs could adsorb metal ions such as Ni^2+^ through metal-affinity coordination [184]. As illustrated in Figure 7C, peptide labeled with His_6_ tags at both ends could trigger the aggregation of Ni^2+^-adsorbed carboxy AuNPs. Cleavage of the peptide into two segments by protease (matrix metalloproteinase) prevented the aggregation of AuNPs. Swartz et al. developed a colorimetric sensor for the detection of histidine-rich proteins based on NTA–Ni^2+^-functionalized AuNPs and AgNPs [187]. In this work, *pf*HRP-II with multiple repeats of AHH and AHHAAD exhibited high affinity toward NTA–Ni^2+^ and could induce the aggregation of NTA–Ni^2+^-functionalized AuNPs in a concentration-and pH-dependent manner. Additionally, AuNPs can catalyze the silver deposition on the nanoparticle surface. Based on this catalytic ability, Cheng et al. reported a scanometric strategy for the determination of matrix metalloproteinases using His_6_-tagged peptide-AuNPs [188]. The metalloproteinases could cleave the specific substrate peptide to release AuNPs from the NTA–Ni^2+^-modified chips. The greyscale signal from the silver enhancement decreased with the reduction of the amount of bound AuNPs.

### 2.5. Others

#### 2.5.1. SERS

SERS can provide the molecular fingerprint information for sensitive chemical andbiological detection. However, most of biomolecules only produce weak SERS response due to their small Raman cross-section and low polarizability. To enhance the sensitivity, silver and gold nanoparticles could be used as the substrates to amplify the SERS signal. NTA–metal complexes modified on the substrate can act as the recognition elements to capture targets from complex samples. The captured target in proximal to the substrate surface would offer a Raman signal. For example, catechol can bind to Fe^3+^ ions with exceptional stability via the coordination interaction. Kaya et al. used NTA–Fe^3+^ complex-modified AgNPs as the substrate for the SERS detection of dopamine in the presence of ascorbic acid [189]. The formed NTA–Fe^3+^–dopamine complexes caused the signal enhancement. In addition, Cao et al. reported the sensitive SERS determination of catecholamine by using NTA–Fe^3+^ complexes-modified polyvinylpyrrolidone-capped AuNPs as the substrates [190]. Li et al. prepared NTA–Ni^2+^ complex-modified AuNPs active substrate to capture histamine via the formation of NTA–Ni^2+^–histamine complex for histamine detection [191].

#### 2.5.2. Chemiluminescence

**Figure 8:**
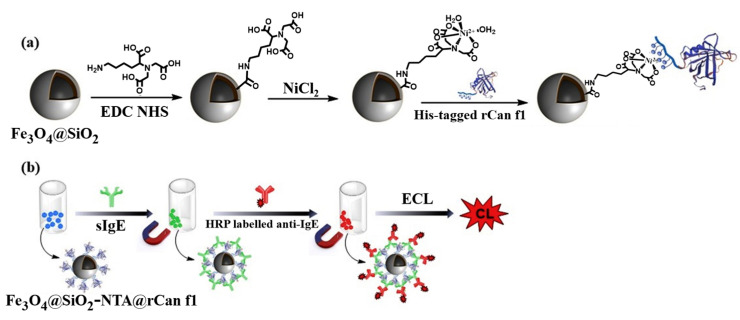
Schematic illustration of (**a**) the synthetic route of Fe_3_O_4_@SiO_2_-NTA and immobilization of rCan f1 via Ni–NTA and His-tag interaction, and (**b**) the immunosensor Fe_3_O_4_@SiO_2_-NTA@rCan f1 for quantitative detection of sIgE in real samples [192]. Copyright 2020 Elsevier.

Chemiluminescence signal can be generated by a redox reaction in which the electronically excited species from a chemical reaction return to the ground state. Based on this principle, Han et al. developed a chemiluminescence immunosensor for the detection of allergen-specific IgE (sIgE) by immobilizing the His_6_-tagged allergens to the NTA-modified MNPs [192]. As shown in Figure 8, NTA was conjugated to the surface of Fe_3_O_4_@SiO_2_ through the amidation reaction and the recombinant His_6_-tagged Can f 1 (rCan f1), which was then adsorbed onto the Fe_3_O_4_@SiO_2_-NTA surface in the presence of Ni^2+^ ions. Next, sIgE in sera from allergic patients specifically captured by Fe_3_O_4_@SiO_2_-NTA@rCan f1 was labeled with HRP-modified anti-IgE. After the magnetic separation, HRP on MNPs surface-catalyzed the chemiluminescence reaction, achieving the quantitative detection of sIgEs.

#### 2.5.3. Immunochromatic Rapid Diagnostic Tests (RDTs)

**Figure 9:**
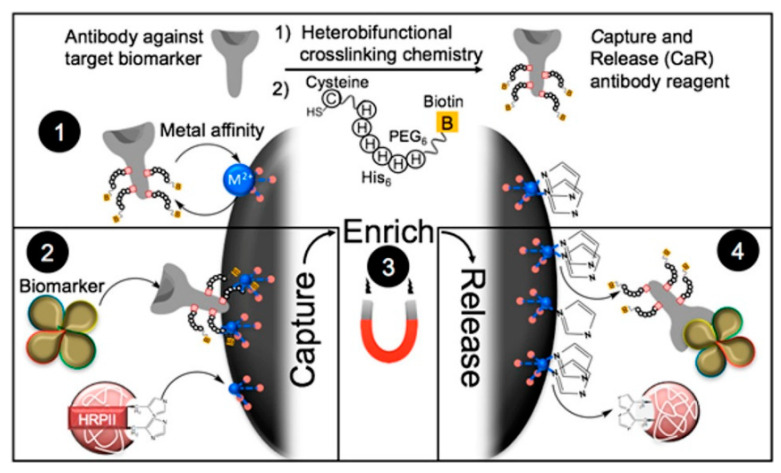
Schematic illustration of conjugation strategy and operation principle for the pLDH and HRPII biomarker enrichment strategy [195]. Copyright 2017 American Chemical Society.

RDTs show the advantages of easy operation, wide applicability, rapid response and low cost [193]. Wright’s group used NTA–Ni^2+^-coated MBs to concentrate the malarial biomarker of histidine-rich protein II (*pf*HRP-II) for enhancing test performances [194]. To realize the detection of low level of infection by multiantigen RDTs, they further developed a magnetically assisted multiplex biomarker enrichment strategy [195]. As shown in Figure 9, the antibodies were first modified with His_6_ tags by maleimide-thiol interaction and then captured by NTA–Ni^2+^-coated magnetic beads. The captured antibody–antigen conjugates could be magnetically purified, concentrated, and then released into a RDT-compatible volume for assay.

Particles in an evaporating colloidal drop can migrate onto the drop’s edge to form a ring on the underlying substrate, which has been widely exploited in the analytical science called as the coffee-ring effect [196]. Wright’s group reported an RDT for the detection of poly-L-histidine (PLH) as a *pf*HRP-II biomimic based on the biomarker-mediated disruption of coffee-ring formation [197]. In this work, *pf*HRP-II promoted the cross-linking between NTA–Ni^2+^-functionalized magnetic particles and indictor particles with red fluorescence. The conjugates were then pulled to the center under a magnetic field. Meanwhile, the indictor particles with green fluorescence were transported to the edge. However, in the absence of *pf*HRP-II, the colocation of both indicator particles at the edge led to a ring with yellow emission with no center signal. However, this method exhibited the shortcomings of low sensitivity, the requirement of an extra magnetic field and a high background signal. To solve those problems, Wright’s group developed a platform for recombinant HRP-II detection using NTA–Ni^2+^ complex-modified gold-plated polystyrene microspheres (AuPS) and NTA–Ni^2+^ complex-functionalized glass [198]. During the coffee ring formation, the conjugate of recombinant HRP-II and AuPS particles moved to the drop edge by binding to the NTA–Ni^2+^ complex-functionalized glass. The non-specific materials could be washed away from the surface.

**Table 1:** Biosensors based on the binding events of NTA–metal complexes.

Detection Techniques	Substrate	Biorecognition Elements	Metal Ions	Target	Linear Range	LOD	Ref.
SPR	NTA-modified gold-coated fiber-optic probe	His_6_-tagged scFv-33H1F7	Co^3+^	PAI-1	3.125~400 ng/mL	0.20 ng/mL	[70]
	Polypyrrole-NTA-modified graphene-gold chip	BiotinylatedCT cholera toxin	Cu^2+^	Anti-CT	4 × 10^−3^~4 ng/mL	4 pg/mL	[101]
	TrisNTA-modified chip	His_6_-tagged S1 protein	Ni^2+^	Anti-SARS-CoV-2 antibody	0.5~96 μg/mL	57 ng/mL	[111]
	TrisNTA-modified chip	His_6_-tagged protein G	Ni^2+^	IgG	0.5~20 μg/mL	47 ng/mL	[113]
	NTA-modified gold-coated fiber-optic probe	His_6_-tagged ADAMTS13	Co^3+^	Anti-ADAMTS13 autoantibodies	1.56~100 ng/mL	0.24 ng/mL	[116]
	NTA-modified electrode	His_6_-tagged receptor	Cu^2+^	Amyloid-beta_16–23_	1 × 10^−3^~1 μM	1.43 nM	[82]
EC	NTA-modified gold electrode	NTA–Cu^2+^	Cu^2+^	Lipopolysaccharide	1 × 10^−4^~0.1 ng/mL	0.1 pg/mL	[84]
	NTA-modified carbon electrode	His_6_-tagged SOD	Ni^2+^	O_2_^•−^	0.1~100 μM	21 nM	[132]
	NTA-modified SPCEs	His_6_-tagged PBP	Co^2+^	Ampicillin	1.3~9.9 ng/mL	0.7 ng/mL	[133]
	NTA-modified Au-coated quartz electrode	His_6_-tagged peptide	Ni^2+^	PKA	0.64~22.33 mU/μL	0.061 mU/μL	[134]
	Polypyrrole-NTA-modified electrode	NH_2_–His_5_-DNA	Cu^2+^	HIV DNA	1 × 10^−6^~10 nM	1 fM	[135]
	Polypyrrole-NTA-modified electrode	His_5_-modified aptamer	Cu^2+^	Thrombin	4.7 × 10^−3^~0.5 nM	4.4 pM	[136]
	Polypyrrole-NTA-modified electrode	His_5_-modified aptamer	Cu^2+^	Bisphenol A	1 × 10^−5^~1μM	10 pM	[137]
	NTA-modified gold electrode	His_6_-tagged Ara h 2	Ni^2+^	Ara h 2 antibody	1~10 pM	1 pM	[138]
	Polypyrrole /NTA-modified electrode	Biotinylated CT B Subunit	Cu^2+^	Anti-CT	1 × 10^−7^~10 μg/mL	0.1 pg/mL	[141]
	NTA-modified gold electrode	NTA		Cu^2+^	0.4~100 μM	10 nM	[144]
	NTA-modified thin-film transistor	NTA		Cu^2+^	0~15 μM	0.51 μM	[145]
FL	Zr–NTA-modified MNPs	EGFP	Ni^2+^	thrombin	3 × 10^−4^~5 × 10^−2^ U/mL	0.3 mU/mL	[151]
	Zr–NTA-modified MNPs	FITC-labeled peptide	Zr^4+^	PKA	0~1 U/μL	0.5 mU/μL	[152]
Color	Carboxy AuNPs	Dual His_6_-tagged peptide	Ni^2+^	MMP-7	3~52 nM	10 nM	[184]
	NTA-modified chip	His_6_-tagged peptide	Ni^2+^	MMP-7	0.1~100 ng/mL	97 pg/mL	[188]
SERS	NTA-modified AgNPs	NTA–Fe^3+^	Fe^3+^	Dopamine	0.5~4 nM	60 pM	[189]
	NTA-modified AuNPs	NTA–Fe^3+^	Fe^3+^	Dopamine, norepinephrine and epinephrine	0.556~10 μM; 0.125~10 μM; 0.2~9.09 μM	Notreported	[190]
	NTA-modified AgNPs	NTA–Ni^2+^	Ni^2+^	Histamine	1~100 μM	1 μM	[191]
CL	Fe_3_O_4_@SiO_2_-NTA	His_6_-taggedCan f 1	Ni^2+^	SpecifcIgE	2.52~10.02 ng/mL	0.35 ng/mL	[192]

Abbreviation: EC, electrochemistry; FL, fluorescence; CL, chemiluminescence; CT, cholera toxin; PKA, protein kinase A; Ara h2, *Arachishypogaea*2; SOD, superoxide dismutase; SPCEs, screen-printed carbon electrodes; PBP, penicillin binding protein; MNPs, magnetic nanoparticles; FITC, fluorescein isothiocyanate; recombinant-enhanced green fluorescence protein; MMP-7, matrix metalloproteinase-7; AuNPs, gold nanoparticles; AgNPs, silver nanoparticles.

## 3. Conclusions

NTA–metal complexes were initially developed as coordination-bonding-based artificial receptors for protein purification. Their pleiotropic merits have facilitated their expanded applications recently as functional and structural agents in multidisciplinary research such as protein engineering, synthetic chemistry and biological analysis. The noncovalent, specific and strong interaction between NTA–metal complexes and His_6_ tags enabled the site-specific and reversible immobilization or labeling of biomolecules, which is helpful to fabricate versatile optical and electrochemical biosensors, especially for point-of-care tests in low-resource settings. For example, NTA–metal complex-functionalized materials, such as cellulose membranes and MBs, have been successfully used to enrich the biomarker concentration for sample preparation, resulting in the enhanced sensitivity of diagnostics. NTA–metal complexes-based affinity techniques provide site-specific, controllable and reversible approaches to immobilize biorecognition elements on detection platforms or nanomaterials under mild conditions without decreasing their functional activities. Moreover, NTA–metal complexes conjugated with other functional species can be used to label His_6_-tagged proteins, and their unique optical, catalytic, electrochemical and magnetic properties have endowed them with signal generation ability in diagnostics.

Despite the successful applications in different research fields, there are still some important challenges to be resolved. For example, the cytotoxicity of NTA–metal-His_6_ tags and their influence on the structure, function and stability of proteins should be carefully investigated even though the site-specific modification of proteins by NTA derivatives have already been widely used for living-cell and single-molecule imaging. In addition, the insufficient fouling resistance ability should be improved when the biosensors are used for the assays of undiluted blood plasma samples. NTA–metal complexes can be coupled with other strategies for signal amplification, such as DNA techniques and enzymes. We believe that the integration of NTA–metal complexes with modern analytical techniques would result in a remarkable boost for the design and implementation of powerful and novel biosensors.

## Data Availability

Not applicable.
